# Supplementary material for: Impact of Service Quality of Low-Cost Carriers on Airline Image and Consumers’ Satisfaction and Loyalty during the COVID-19 Outbreak
Source: Int J Environ Res Public Health. 2021 Dec 22;19(1):83. doi: 10.3390/ijerph19010083 (PMC8750909; doi:10.3390/ijerph19010083)
Supplement: Supplementary file 1 [file ijerph-19-00083-s001.zip › ijerph-1489284-supplementary.pdf]

Table S1. Group-based differences of different constructs based on the Demographic and travel-related characteristics.

Parameter	Category	Service Quality	Reliability	Assurance	Empathy	Tangible	Responsiveness	Satisfaction	Image	Loyalty
Gender	Male	3.67 (3.07-4.20)	3.83 (3.17-4.17)	3.33 (2.67-4.00)	4.00 (3.00-4.00)	4.00 (3.00-4.50)	3.50 (2.50-4.50)	3.50 (3.00-4.50)	4.00 (3.00-4.33)	3.50 (3.00-4.50)
	Female	3.67 (2.47-4.25)	3.67 (2.17-4.33)	3.33 (2.33-4.00)	3.75 (2.50-4.50)	3.75 (2.50-4.50)	4.00 (2.50-4.50)	3.50 (2.50-4.50)	3.67 (2.67-4.67)	3.00 (2.00-4.00)
	**p***	**0.492**	**0.246**	**0.365**	**0.792**	**0.422**	**0.441**	**0.583**	**0.424**	**0.012**
Age	Under 18	4.00 (2.63-4.50)	3.83 (2.58-4.33)	4.00 (2.83-5.00)	4.00 (2.50-4.75)	3.50 (2.50-4.75)	3.50 (2.75-5.00)	3.00 (2.50-4.25)	4.00 (2.33-4.50)	2.00 (1.00-4.00)
	18-24	3.53 (2.33-4.27)	3.50 (2.00-4.46)	3.00 (2.33-4.00)	3.50 (2.50-4.50)	4.00 (2.00-4.50)	4.00 (2.00-4.50)	3.50 (2.50-4.50)	3.67 (2.67-4.67)	3.25 (2.00-4.50)
	25-34	3.80 (3.40-4.20)	4.00 (3.50-4.29)	3.67 (2.75-4.00)	4.00 (3.00-4.50)	4.00 (3.00-4.50)	3.75 (3.00-4.50)	4.00 (3.50-4.00)	3.83 (3.00-4.67)	3.50 (3.00-4.38)
	35-44	3.60 (3.07-4.13)	3.83 (3.33-4.17)	3.33 (2.67-4.00)	4.00 (3.00-4.50)	3.50 (3.00-4.50)	3.50 (3.00-4.50)	3.50 (3.00-4.00)	3.67 (3.00-4.00)	3.50 (3.00-4.00)
	> 45	3.67 (2.80-4.20)	3.83 (2.67-4.17)	3.33 (2.67-4.00)	4.00 (3.00-4.00)	3.50 (3.00-4.50)	4.00 (1.50-4.50)	3.50 (2.50-4.50)	3.67 (3.00-4.67)	3.50 (2.50-4.00)
	**p¥**	**0.636**	**0.413**	**0.431**	**0.655**	**0.963**	**0.97**	**0.196**	**0.69**	**0.231**
Number of previous trips via LCCs	Just once	3.80 (3.20-4.20)	3.83 (3.00-4.33)	3.33 (2.67-4.00)	4.00 (3.00-4.50)	3.50 (3.00-4.50)	4.00 (3.00-4.50)	3.50 (2.50-4.00)	3.67 (3.00-4.67)	3.50 (2.50-4.50)
	2 times	3.63 (2.53-4.33)	3.67 (2.33-4.33)	3.33 (2.33-4.33)	3.50 (2.63-5.00)	4.00 (2.50-4.50)	3.75 (2.00-5.00)	3.50 (2.50-4.50)	3.67 (2.67-4.33)	3.50 (2.50-4.38)
	3 or more	3.27 (2.33-4.18)	3.50 (2.00-4.33)	3.00 (2.33-4.00)	3.50 (2.50-4.38)	3.50 (2.00-4.50)	3.50 (2.00-4.50)	3.50 (2.63-4.50)	3.67 (2.33-4.33)	3.00 (2.00-4.00)
	**p¥**	**0.073**	**0.068**	**0.060**	**0.104**	**0.739**	**0.098**	**0.954**	**0.351**	**0.080**
Purpose of the most recent trip	Business	3.60 (2.67-4.20)	3.83 (2.50-4.33)	3.00 (2.33-4.00)	3.50 (2.50-4.50)	3.50 (2.50-4.50)	3.50 (2.00-4.50)	3.50 (3.00-4.50)	3.67 (2.33-4.33)	3.50 (2.00-4.00)
	Leisure	3.73 (2.53-4.27)	3.83 (2.17-4.33)	3.67 (2.67-4.33)	4.00 (2.50-4.50)	4.00 (2.50-4.50)	4.00 (3.00-5.00)	3.50 (2.50-4.50)	4.00 (3.00-4.67)	3.50 (2.50-4.50)
	Both Business and Leisure	3.40 (3.40-3.40)	3.17 (3.17-3.17)	3.33 (3.33-3.33)	3.00 (3.00-3.00)	4.50 (4.50-4.50)	3.50 (3.50-3.50)	2.00 (2.00-2.00)	4.67 (4.67-4.67)	4.50 (4.50-4.50)
	Education	2.83 (2.53-4.33)	2.75 (1.88-4.46)	3.17 (3.00-4.00)	3.50 (2.63-4.38)	3.00 (2.00-4.00)	2.75 (2.00-4.88)	3.25 (2.00-4.00)	3.33 (2.50-4.58)	3.25 (2.50-4.38)
	Hajj/Umrah	3.73 (3.07-4.02)	3.67 (3.29-4.00)	3.33 (2.33-4.00)	4.00 (3.50-4.50)	3.75 (3.00-4.50)	3.50 (2.50-4.50)	3.50 (2.50-4.00)	3.67 (2.67-4.33)	3.50 (2.50-4.50)
	**p¥**	**0.862**	**0.868**	**0.619**	**0.483**	**0.513**	**0.465**	**0.323**	**0.450**	**0.593**

* results were based on a Mann-Whitney U test; ¥ results were based on a Kruskal Wallis Test.
